# A Revolution in Treatment for Hepatitis C Infection: Mitigating the Budgetary Impact

**DOI:** 10.1371/journal.pmed.1002031

**Published:** 2016-05-31

**Authors:** Elliot Marseille, James G. Kahn

**Affiliations:** 1 Health Strategies International, Oakland, California, United States of America; 2 Philip R. Lee Institute for Health Policy Studies, University of California, San Francisco, San Francisco, California, United States of America

## Abstract

In a Perspective accompanying Hill and colleagues, Elliot Marseille and James Kahn compare the history of pricing and availability of ART for HIV with that of the new HCV drugs and discuss strategies for providing treatment in LMIC even in the face of high costs.

The new generation of direct-acting antiviral drugs for hepatitis C (HCV) are extraordinarily effective, safe and well tolerated. With a relatively short 12-week course of treatment costing approximately US$84,000 in the United States, they are also extraordinarily costly [1].

In their study in *PLOS Medicine*, Suzanne Hill and colleagues address important economic implications of the new HCV therapies in 26 Organisation for Economic Cooperation and Development (OECD) member countries plus four low- and middle-income countries (LMICs). Given medicine prices specific to each country and the recommended 12-week treatment, the authors calculated total drug costs, which they compared with the total pharmaceutical expenditure (TPE) and the average annual wage in each country. By these measures of affordability, the authors argue that the new drug regimens “…threaten the sustainability of health systems in many countries and prevent large scale provision of treatment.” They call for fairer prices that would allow countries to invest in more treatment and thus lower the burden of HCV.

Lower prices would ease the budgetary impact of these drugs and accelerate access. However, we believe that even with the current price structure, in each country the budgetary impact need not be as severe as suggested. In this Perspective, we compare the history of the pricing and availability of antiviral therapy for HIV with that of HCV. Elucidating points of similarity, and particularly differences, may aid in understanding the challenges and opportunities associated with the HCV therapies.

Both HIV anti-retroviral therapy (ART) and the new HCV therapies are highly effective, revolutionizing the responses to these diseases. While no cure, ART famously had the “Lazarus effect” of bringing patients back from the brink of death. With subsequent refinement of the regimens, patients may live as long as their HIV-negative counterparts and with a tolerable side-effect profile [2,3]. Similarly, the new generation of HCV treatments are very effective, with cure rates of 95%. The other similarity is the very high price. At an annual cost of US$12,000 in the early days of ART, these prices ensured that ART could not be implemented at scale in LMICs, even with assistance from donors. Similarly, at a list price for HCV therapies of about US$84,000 in high-income countries, the onerous short-term budgetary impact guarantees that the new therapies will not be immediately available to all those who might benefit in rich countries, let alone LMICs. But here the similarities end, and the differences are more illuminating.

## Multi-tiered Pricing from the Start

Starting in 1997, the pharmaceutical industry resisted calls by activists for lower ART prices in LMICs, citing trade agreements, law, and the long-standing practice of having one global price for pharmaceuticals under patent. A milestone was reached in February 2001, when the Indian pharmaceutical company Cipla offered ART for US$350 per person-year [4]. Depending on specific regimen, this is a price reduction of about 97%. Since that time, prices have incrementally decreased, to about US$200 per person-year, with first-line regimens approaching 1.5% of the original US$12,000 annual cost [5]. This history stands in stark contrast to the very rapid discounts in HCV drug prices for LMICs. It is possible that the manufacturers of the new HCV drugs, contemplating the long and rancorous history of the ART price wars, determined not to repeat it. Shortly after the US Food and Drug Administration (FDA) approved sofosbuvir and ledipasvir for treatment of HCV in October 2014 [6], Gilead announced a price of US$900 for a 12-week treatment in highly endemic Egypt, a reduction of 99% compared with US prices. Comparable prices for Pakistan [7] were announced in 2015. This discount is similar to the relative reduction in the annual cost of ART for HIV in LMICs. The difference is the rapidity of achieving it. Gilead plans to include 101 LMICs for sofosbuvir access via generic licensing partnerships with Indian manufacturers [8].

## Cure Not Treatment

HCV drugs cure at very high rates in 3 months, while HIV infection remains a chronic condition even with optimal treatment. Thus, while the first-year cost for treating HCV is much higher than for HIV, the two interventions have the same aggregate discounted drug cost in about 4–5 years, in both high- and low-income countries. Our point is not that the current prices are correct or fair, but simply that they are lower than the cost of another important global health intervention that has been implemented at large scale.

## Downstream Savings

The savings to the medical system in averted future costs of liver complications were excluded from the assessment developed by Hill and colleagues. However, studies of the cost-effectiveness of HCV therapies in the US suggest that these benefits are substantial and can help finance HCV treatment [9].

## Duration of Initial Costs

Despite the discounts offered in both LMICs and OECD countries, the short-term impact of HCV treatment on budgets of health care payers and individuals may limit access. However, there are two mitigating factors. First, once the backlog of prevalent cases is treated, the budgetary impact drops dramatically, as only the relatively few incident cases need be treated. Thus, while fiscally disruptive if all HCV-infected persons were immediately put on treatment, that disruption would last only 1 year. Second, treating everyone in year 1 is implausible. The process of identifying cases, limits to health care system capacity, and patient preferences all suggest a multi-year catch-up process. For these reasons, the fiscal burden expressed as a percent of TPE or as a portion of the average annual wage would be much less than the maximum burden as presented in Hill and colleagues’ article.

One solution, then, is to spread the upfront cost of treatment over several years. Not everyone is eager to be treated, especially the asymptomatic for whom delay may be less harmful. Beyond this, there are options for phasing in treatment gradually by equity concerns, i.e., treating those with lower access to care first, or by disease stage. Our US-based analysis found that while treating all patients in fibrosis stages 1–4 was cost-effective, initiating treatment in stages 3 and 4 was more cost-effective and would reduce total net treatment costs in the US by about one-third (from US$89,804 to US$60,906) per individual with chronic hepatitis C [9]. A combination of equity and disease stage criteria can match phase-in plans to different countries’ budgets and political will.

This is not an argument against political activism or additional market competition and innovation to reduce HCV drug prices—indeed, a recent report of a pilot study suggests that a six-week drug regimen may be sufficient to cure acute HCV infection [10]. All else equal, lower drug cost means better access to life-saving therapy. However, there is no need to postpone treatment pending these developments. It is in each country’s capacity, and without disruptive budgetary impact, to start treating many of those most in need of care now, and to extend coverage to all over the succeeding few years.

## Abbreviations

ART: anti-retroviral therapy
FDA: Food and Drug Administration
HCV: Hepatitis C
LMIC: low- and middle-income country
OECD: Organisation for Economic Cooperation and Development
TPE: total pharmaceutical expenditure
